# Enhancing the yield and berry color of crimson seedless table grape variety (*Vitis vinifera* L.) via zinc oxide nanoparticles integrated with molasses

**DOI:** 10.1186/s12870-026-08837-8

**Published:** 2026-05-09

**Authors:** Mina S. F. Samaan, Mohamed A. Nasser, Ahmed Abou El-Yazied, Sabry Soliman, Shaimaa Ismail, Mohamed K. Abou El-Nasr

**Affiliations:** Department of Horticulture, Faculty of Agriculture, Ain Shams University, 68-Hadayek Shoubra, Cairo, 11241 Egypt

**Keywords:** Crimson seedless, grapevine, ZnO, NPs, berry coloration, anthocyanin

## Abstract

**Background:**

Crimson Seedless is a globally important table grape cultivar valued for its color, marketability, and nutritional quality. However, enhancing berry coloration and biochemical quality remains a major challenge, especially under fluctuating environmental conditions. In this context, the individual application of nanoparticles or molasses as natural organic stimulants may offer an effective. This study hypothesizes that foliar application of zinc oxide nanoparticles (ZnO NPs) and molasses, either individually or in combination, would increase yield, berry coloration, and antioxidant-related biochemical parameters compared with those of the untreated control. The experiment included eight treatments applied to five grapevines per treatment via a randomized complete block design (RCBD) over two consecutive seasons (2023–2024), and the effects of ZnO NPs at 10, 50, and 100 mg L^− 1^, applied alone or in combination with molasses at 1.5 cm³ L^− 1^, on productivity, cluster traits, anthocyanin accumulation, and antioxidant-related enzymes were evaluated.

**Results:**

In terms of productivity, cluster quality, and berry coloration, the treatment with ZnO NPs plus molasses followed closely by ZnO NPs alone surpassed the nontreated vines. The 10 mg L^− 1^ ZnO NPs + molasses treatment was superior for most of the studied parameters. For example, the total yield increased by approximately 8.9–11.4%, the total soluble solid (TSS) content increased by 48.4–43.8%, the firmness increased by 8.1–12.0%, and the acidity decreased by 16.3–15.4% compared with those of the control treatment in both seasons. Additionally, the same treatment resulted in the greatest increase in anthocyanin content (104.3–73.4%) and PAL activity (48.4–58.1%) compared with those of the control in both seasons.

**Conclusion:**

The application of 10 mg L^-1^ ZnO NPs + 1.5 cm^3^ L^-1^ molasses once at the veraison stage increased the grape yield, color, and biochemical quality. While a single-stage application was effective, further studies could explore multistage applications or alternative timings to optimize the results.

**Graphical Abstract:**

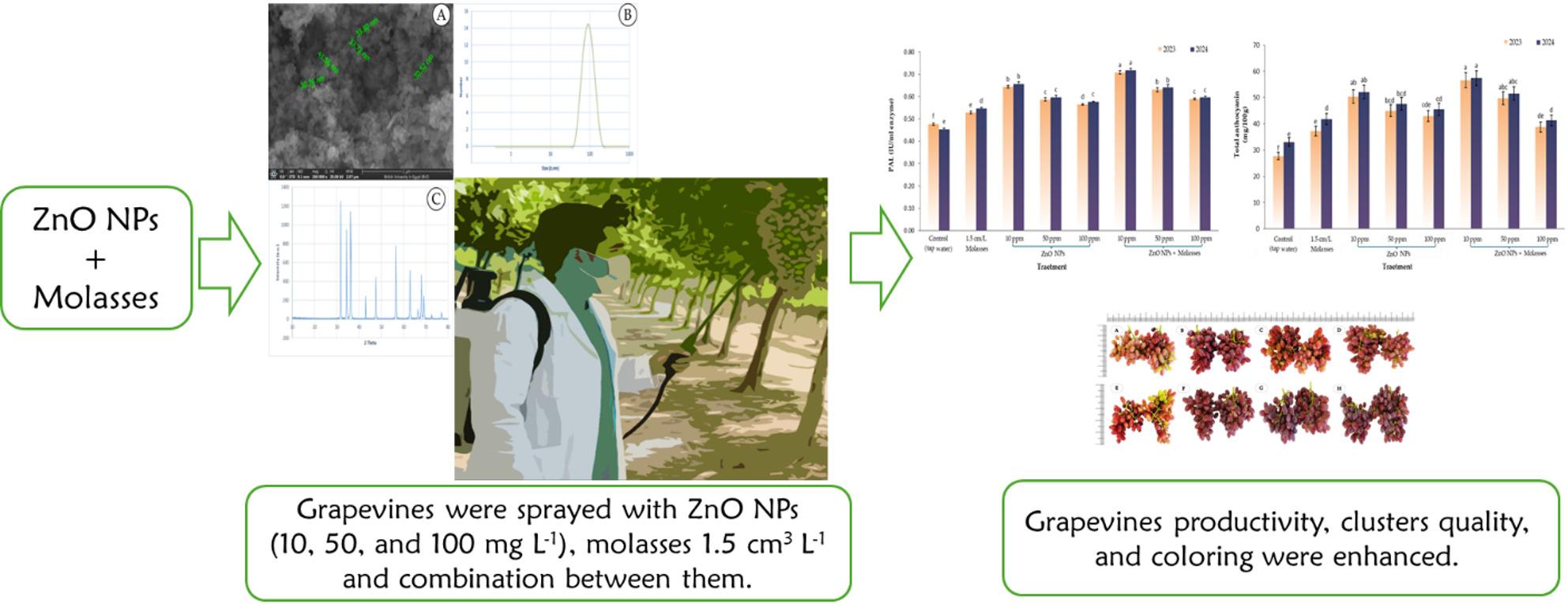

## Background

To promote sustainability, agricultural practices are being developed to reduce environmental degradation and ensure long-term food production [1–5]. The excessive utilization of chemical fertilizers has led to environmental pollution, greenhouse gas emissions, and disruption of ecosystems [6–8]. Therefore, more sustainable strategies are urgently needed to improve crop productivity while reducing chemical inputs. Among fruit crops, grapevines are particularly sensitive to environmental changes that affect berry coloration a major quality determinant [9–11]. Grapevine (*Vitis vinifera* L.) is a deciduous fruit tree belonging to the Vitaceae family. It is native to Europe and widely cultivated in different climatic regions [12]. In Egypt, grapevine production in 2023 was estimated at more than 1.9 million tons from an area of approximately 83.2 thousand hectares, with an average yield of 23.1 tons per hectare [13]. One of the main problems with fruit quality is inadequate berry color development, which is considered a key factor in determining the marketability of fruits [14]. Recent advances in nanotechnology have emerged as promising tools to address such challenges by improving nutrient use efficiency and stress resilience. Nanoparticles (NPs) play essential roles in modulating physiological and biochemical processes, including photosynthesis, nutrient uptake, and secondary metabolite accumulation [15, 16]. In grapes, nanomaterials have been reported to stimulate anthocyanin biosynthesis, delay oxidative stress, and improve berry coloration under suboptimal climatic conditions [17, 18]. Their small size and high reactivity allow them to efficiently penetrate plant tissues, thereby influencing the expression of key genes involved in pigment formation and sugar metabolism [19]. In addition to nanotechnology, natural organic additives such as molasses can increase nutrient uptake and microbial activity, resulting in biobased synergy.

Integrating nanotechnology with sustainable viticulture practices could provide a promising strategy for improving Crimson Seedless grape quality while reducing reliance on conventional chemical inputs. The application of nanoparticles at optimal concentrations may increase berry color and market value; however, their environmental impact and residue dynamics require further investigation [17, 20]. Crimson Seedless is one of the most widely grown and commercially important cultivars. It is a late-maturing red variety with crispy berries, desirable fruit quality attributes, and good natural flavor [21–23]. Insufficient development of berry coloration, one of the primary indicators of fruit quality, is a challenging problem [24, 25]. The characteristic pigmentation of this cultivar is attributed to anthocyanins, which are affected by climate and daily temperature variations during the ripening stage [2]. There are several solutions for irregular coloration in clusters, including the application of chemical compounds, phytohormones or nanomaterials that influence regulation and improve coloration, as observed when Crimson Seedless grapes are sprayed with ZnO NPs [14].

Zinc (Zn) is an essential micronutrient that is present in small amounts. It plays a vital role in numerous biological processes, including enzyme activation, nucleic acid and protein synthesis, membrane stabilization, pathogen defense, auxin regulation, chlorophyll formation, and carbohydrate metabolism [26, 27]. Zinc plays a fundamental structural and catalytic role in numerous enzymes and transcription factors involved in plant metabolism [28, 29]. In grapevine, anthocyanin biosynthesis is regulated through the phenylpropanoid pathway, in which phenylalanine ammonia-lyase (PAL) acts as a key entry enzyme [30]. The subsequent steps involve enzymes such as chalcone synthase (CHS) and UDP-glucose: flavonoid 3-O-glucosyltransferase (UFGT) [31], which are critical for pigment accumulation during ripening.

The unique characteristics of materials at the nanometric scale, including their small size and large surface area, broaden their scope of use in various advanced fields of agriculture, leading to increased interest in their application [19, 32–35]. Nanomaterials are increasingly being utilized in agriculture to improve plant biomass. Micronutrient nanoparticles are more suitable for foliar application than for soil application because of their ease of application, reduced risk of toxicity due to micronutrient accumulation, and prevention of soil fixation [36]. In addition, nanoparticle-based foliar applications may provide an important adaptive advantage by enhancing plant tolerance to abiotic stress conditions, thereby contributing to yield stability and fruit quality under fluctuating environmental conditions [37]. Zinc oxide nanoparticles (ZnO NPs) have recently gained prominence in numerous sectors because of their high biological activity, stability [38], and potential to modulate the activity of antioxidant enzymes, increase nutrient utilization, and increase photosynthetic performance [39]. According to the US Food and Drug Administration, ZnO NPs are considered generally recognized as safe and more beneficial for use in a wide range of consumer products [40, 41]; however, the safety of ZnO nanoparticles may vary depending on their physicochemical properties, application rates, and environmental conditions. Several investigations have reported that foliar application of ZnO NPs at low doses to plants plays an important role in regulating plant growth and metabolism [42, 43]. ZnO NPs increase the growth and productivity of many plants, such as coffee [44] and grape [14, 45], regulate biosynthesis, increase fruit quality [46], and act as cofactors for the activity of many enzymes [47–49].

Molasses is a major byproduct of the sugar processing and fermentation industries [50]. Molasses is a viscous dark brown liquid with a high content of recalcitrant compounds, such as melanoidins, and possesses unique properties [51–53]. Molasses can increase nutrients and act as soil conditioners and biofertilizers [54]. Additionally, when applied to the soil, it improves the physical composition of the soil, the biological activity of beneficial microorganisms, and crop productivity [55]. Plants treated with molasses presented significant increases in growth, photosynthetic pigments, productivity, and quality in spinach [56], onion [57], sugar beet [58], and wheat [59]. Although molasses is widely used as a soil amendment, its foliar application may also exert physiological effects [60]. Molasses contains readily available sugars [61], organic acids, amino acids, and trace elements [62] that can be absorbed through leaf surfaces. During fruit ripening, exogenous sugars may act as metabolic signals that increase carbohydrate metabolism and promote the biosynthesis of secondary metabolites, including anthocyanins [63–65]. Additionally, foliar-applied molasses may support energy-demanding biosynthetic pathways by providing carbon substrates, thereby complementing the enzymatic activation induced by ZnO NPs.

To date, studies on grapevines have evaluated primarily ZnO NPs or organic amendments independently. However, the combined foliar application of ZnO NPs and molasses as an integrated nano-organic strategy has not been systematically investigated in Crimson Seedless grapevines under field conditions. This study hypothesizes that foliar application of ZnO NPs in combination with molasses significantly enhances grapevine productivity and fruit quality compared with individual applications or untreated vines by increasing physiological and biochemical processes, including increased antioxidant activity and increased anthocyanin biosynthesis, thereby improving berry coloration in Crimson Seedless grapes under field conditions.

## Materials and methods

### ZnO NPs characterization

Zinc oxide nanoparticles (ZnO NPs) were purchased from US Research Nanomaterials, Inc., and the product numbers were as follows: US1019F, CAS#: 13463-67-7. The nanomaterial sample was characterized through several tests according to [66], including the morphology of the ZnO NPs, which was characterized by scanning electron microscopy (SEM, Thermo Scientific™ Q250, USA). The differences between the SEM-derived particle size and DLS measurements were attributed to particle aggregation and hydration effects in the suspension. The particle size distribution and zeta (ζ)-potential were measured via a particle analyzer (Zetasizer Nano, Malvern, UK). The crystallographic phase pattern of the ZnO NPs was analyzed via X-ray diffraction (X’Pert PRO, PANalytical, Netherlands).

### Molasses characterization

Molasses, a byproduct of sugar beet processing, was purchased from Delta Sugar Co., Egypt. The beet molasses used in this experiment was a viscous, dark-brown liquid rich in sugars and minerals. Its chemical composition is presented in Table 1.

**Table 1:** Chemical composition of molasses from beet sugar processing

Parameters	Results	Parameters	Results
Dynamic viscosity (cP)	1309.2	N (%)	1.70
pH	8.1	P (%)	0.12
Brix (%)	79.00	K (%)	4.10
Total sugar (%)	52.34	Ca (%)	0.20
Purity (%)	60.00	Mg (%)	0.08
Ash (%)	9.90	Na (%)	1.80

### Plant material and treatments

In two consecutive seasons (2023 and 2024), this experiment was conducted on a private vineyard (Elgandour Farm, latitude: 30° 17’ 27.0” N and longitude: 30° 32’ 20.2” E) located at the Cairo Alex Desert Road, K78, Egypt. The landowner gave permission to collect samples of Crimson seedless grapes from the vineyard. The region has a temperate climate, with an average annual temperature of 21 °C, an average diurnal temperature variation of 11.3 °C, a yearly sunshine duration of approximately 3538 h, and an average annual rainfall of 144 mm. The experiment was conducted via a randomized complete block design (RCBD) on six-year-old Crimson Seedless grapevines, which were grafted on Richter 110 rootstock and selected for uniformity; these plants were planted in sandy soil with drip irrigation. The distance between plants was 2 × 3 m in the Parron training system. Every grapevine was exposed to identical horticultural procedures that were advised on the basis of the conditions of the vineyard.

Grapevines were sprayed with tap water (control), molasses at 1.5 cm^3^ L^− 1^, ZnO NPs at three concentrations [10, 50, and 100 mg L^− 1^], or a combination of molasses at 1.5 cm^3^ L^− 1^ + ZnO NPs at 10 mg L^− 1^, molasses at 1.5 cm^3^ L^− 1^ + ZnO NPs at 50 mg L^− 1^, or molasses at 1.5 cm^3^ L^− 1^ + ZnO NPs at 100 mg L^− 1^. Before application, nanoparticle suspensions were prepared in distilled water and sonicated for 30 min in an ultrasonic bath to ensure homogeneous dispersion. Twenty liters of spraying solution were prepared with the addition of carboxymethylcellulose (CMC) [1%] at a rate of 4 ml per 20 L for each treatment to help form a constant suspension. The same CMC concentration was included in all the treatments, including the control, to avoid any confounding physiological effects. The grapevines were sprayed in the early morning via a 20 L hand-held sprayer, and the grapevines were sprayed until runoff, with an average application volume of approximately 4 L per vine, depending on the canopy size. The treatments were applied once at the veraison stage in each season. The spraying was carried out during the month of July, with the average maximum temperature being 38 °C in both seasons, the average minimum temperature being 21 °C in 2023 and 24 °C in 2024, and the relative humidity ranging from 30% to 50% in both seasons.

### Grapevine productivity

All clusters in each treatment were harvested in mid-July in both seasons when the red color covered all bunch berries. The total yield per vine was calculated by counting and weighing the clusters on each grapevine.

$$\begin{aligned}\text{Total yield per grapevine} &= \text{number of clusters per vine}\\& * \text{average weight of clusters}\end{aligned}$$

### Physicochemical properties evaluation of clusters

The total soluble solids in berry juice obtained via manual pressing were measured via a refractometer (PZO - RR13, Poland). For titratable acidity (TA), fruits were cut into pieces to make a homogeneous mixture. After that, a sample of 5 g was taken and then homogenized for 40 s in a blender. TA was determined in grams of tartaric acid per 100 ml of juice according to [67]. To determine the firmness of the berries, a GÜSS Fruit Texture Analyzer (FTA) was used, and the maximum force required was recorded in g/mm.

### Biochemical components of clusters and PAL enzyme activity

#### Determination of total anthocyanin

The total anthocyanin content was determined according to the methods of [68, 69]. Extraction was performed at 4 °C in darkness to prevent pigment degradation. One gram of fresh berry skin was soaked for 24 h. in acidic alcohol (1 M HCl/methanol at 15:85 v/v) to extract the pigment under dark conditions, and then the samples were measured via a spectrophotometer at a wavelength of 530 nm. The values are expressed as total anthocyanin (mg/100 g FW). All the determinations were performed in triplicate.

#### Assays of PAL activity

Enzyme activity (IU/ml enzyme) was estimated according to [70]. In brief, 1 g of berry skin tissue was ground in 4 mL of extraction phosphate buffer (1 M, pH 7). The homogenates were centrifuged under cooling for 15 min. The supernatant was collected and used for the assays of enzymatic activities. The estimate is made by adding 1 ml of borate buffer (pH 8.8, 0.1 M), adding 1 ml of 12 mM L-phenylalanine, and then adding 0.8 ml of the enzymatic extract. The increase in the absorbance at 290 nm was monitored because of the formation of cinnamic acid. Enzyme activity was calculated and expressed as IU mL⁻¹ of crude enzyme extract, without normalization to protein content.

#### Color analysis

Berry skin color was quantified via digital image-based color analysis following standard colorimetric procedures. For each replicate, 10 as uniform as possible berries were randomly selected, resulting in 50 berries per treatment. Images were captured via a high-resolution digital camera (24 MP) under controlled lighting conditions (5500 K LED light source) with a uniform black background. All the images were analyzed via Python (version 3) with the scikit-image library. Berry regions were segmented via threshold-based masking to isolate the skin area from the background. The extracted regions were converted from the RGB space to the CIE-Lab color space. For each berry, the mean values of L* (lightness), a* (red–green component), and b* (yellow–blue component) were calculated from all the pixels within the segmented skin area. The average values per replicate were then computed. The color index for red grapes (CIRG) was computed according to Carreño [71].

#### Statistical analysis

The experiment was arranged in a randomized complete block design (RCBD); each treatment was conducted with five replicates, and each replicate included one vine, resulting in 5 vines per treatment. All the statistical analyses of the different traits were performed via SPSS software (version 20). Differences among treatments were tested via Duncan’s multiple range test [72]. A biplot of principal component analysis (PCA) was generated via standardized data (z-score normalization) to eliminate scale effects among variables. Data standardization was performed according to the formula $\:Z=\frac{X-\mu\:}{\sigma\:}$, where: Z represents the standardized variable, X is the original variable value, µ is the mean of the variable, and σ is its standard deviation. The PCA analysis was conducted via XLSTAT (version 2018.1). The input matrix consisted of treatments data for all measured parameters. PCA was conducted separately for each season. Heatmaps were constructed to summarize differences among treatments for the measured parameters via min–max scaling to visualize relative differences among treatments in Microsoft Excel (Office 365). Pearson correlation analysis was performed to evaluate the relationship between PAL activity and anthocyanin content via all treatment means (*n* = 8). Statistical significance was determined at *p* ≤ 0.05.

## Results

### ZnO NPs characterization

**Fig. 1:**
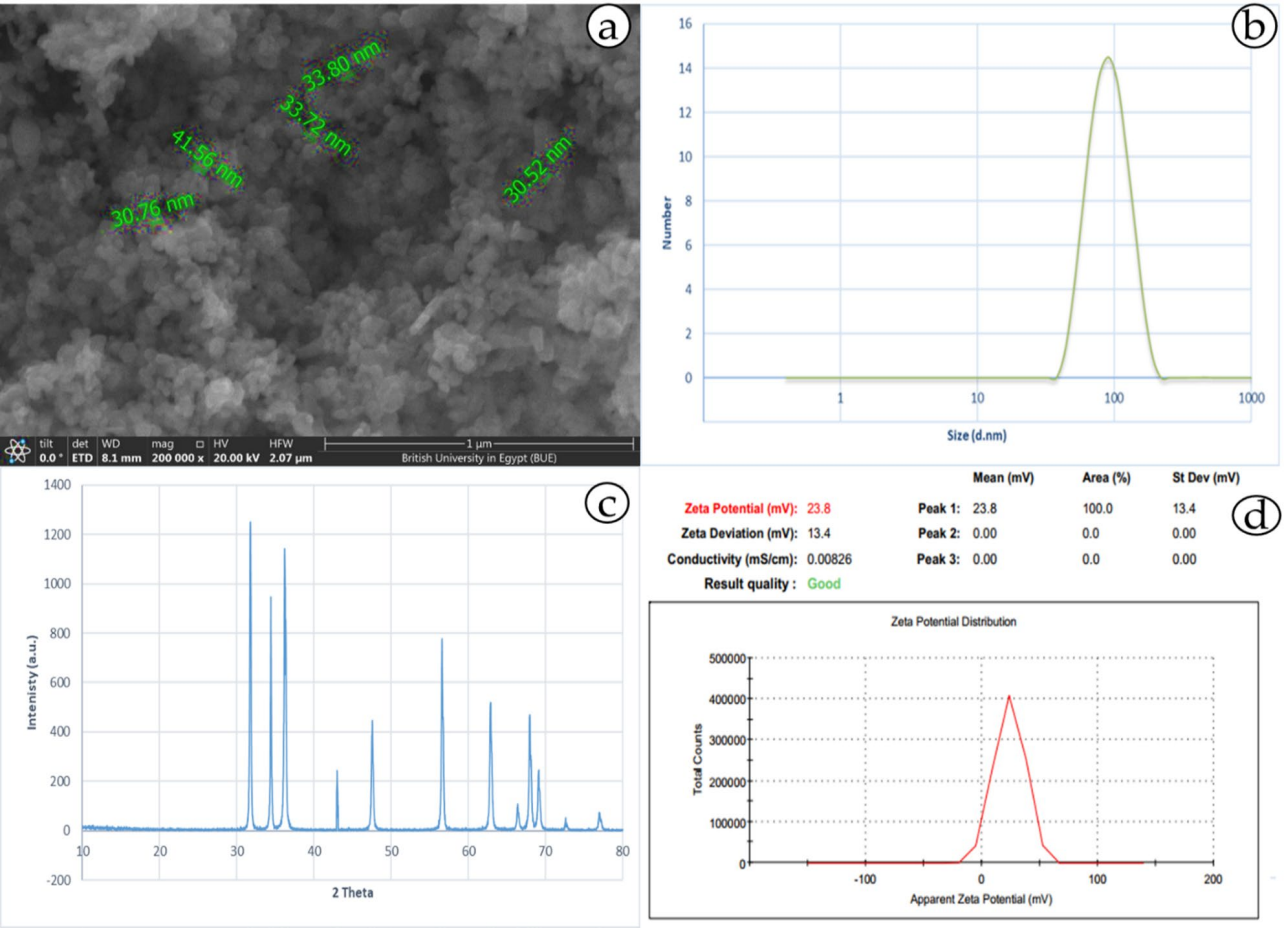
Examination of ZnO NPs: **(A)** SEM image, **(B)** size distribution, **(C)** X-ray diffraction analysis, and **(D)** zeta potential

The morphology of the ZnO nanoparticles was characterized via FESEM images (Fig. 1A), which revealed a predominantly spherical morphology. Dynamic light scattering (DLS) revealed an average particle size of 91.3 nm, as shown in Fig. 1B. X-ray diffraction (XRD) analysis confirmed the crystalline phase of the nanoparticles (Fig. 1C), and the XRD pattern of the sample revealed the formation of ZnO. A comparison of their XRD patterns with the standard pattern of ZnO revealed that they were consistent with the characteristic peaks of the ZnO crystal. The ζ-potential was measured to be 23.8 mV via electrophoretic light scattering (Fig. 1D).

### Grapevine yield

Our findings indicated that foliar application of ZnO NPs and molasses influenced several parameters of grape clusters, including cluster weight and total yield per vine, as shown in Table 2. The results clearly demonstrated the significant (*p* ≤ 0.05) effect of the applied treatments on the average weight of the grape clusters during both the 2023 and 2024 seasons. The 10 mg L^− 1^ ZnO NPs + molasses treatment resulted in the highest cluster weights (480.53 and 482.53 g) in both seasons, while the control treatment resulted in the lowest cluster weights (441.07 and 433.27 g) in both seasons. Other treatments, such as ZnO NPs alone or molasses alone, had intermediate effects. Compared with untreated grapevines, foliar spraying with low concentrations of ZnO NPs alone or in combination with molasses significantly increased the total yield of grapevines. The highest value was observed under 10 mg L^− 1^ ZnO NPs + molasses, with values of 14.42 and 14.48 kg/grapevine in both seasons, reflecting 8.9 and 11.4% increases over those of the control in both seasons, respectively.

**Table 2:** Effect of ZnO nanoparticles and molasses foliar application on the cluster weight and total yield of Crimson seedless grapes during two seasons (2023 and 2024)

Parameter	Cluster weight (g)	Total yield/grapevine (kg)	Cluster weight (g)	Total yield/grapevine (kg)
Treatments	The first season (2023)		The second season (2024)	
Control (tap water)	441.07 ^c^ ± 1.70	13.23 ^c^ ± 0.05	433.27 ^d^ ± 4.90	13.00 ^d^ ± 0.15
1.5 cm/L Molasses (M)	452.80 ^bc^ ± 4.10	13.58 ^bc^ ± 0.12	453.00 ^bcd^ ± 11.02	13.59 ^bcd^ ± 0.33
10 mg L^− 1^ ZnO NPs	467.67 ^ab^ ± 4.20	14.03 ^ab^ ± 0.13	466.13 ^abc^ ± 6.16	13.98 ^abc^ ± 0.18
50 mg L^− 1^ ZnO NPs	457.53 ^bc^ ± 2.97	13.73 ^bc^ ± 0.09	462.20 ^abc^ ± 3.35	13.87 ^abc^ ± 0.10
100 mg L^− 1^ ZnO NPs	441.53 ^c^ ± 5.89	13.25 ^c^ ± 0.18	441.87 ^cd^ ± 2.37	13.26 ^cd^ ± 0.07
10 mg L^− 1^ ZnO NPs + M	480.53 ^a^ ± 10.47	14.42 ^a^ ± 0.31	482.53 ^a^ ± 7.05	14.48 ^a^ ± 0.21
50 mg L^− 1^ ZnO NPs + M	470.87 ^ab^ ± 7.83	14.13 ^ab^ ± 0.24	468.73 ^ab^ ± 6.38	14.06 ^ab^ ± 0.19
100 mg L^− 1^ ZnO NPs + M	458.86 ^bc^ ± 10.19	13.77 ^bc^ ± 0.31	466.33 ^abc^ ± 14.28	13.99 ^abc^ ± 0.43

Means in each column with the same letter(s) are not significantly different at the 5% level

### Physicochemical properties

According to the results shown in Table 3, foliar application of ZnO NPs, either alone or in combination with molasses, significantly affected the total soluble solids (TSS), acidity percentage, TSS/acid ratio, and berry firmness of Crimson Seedless grapes in both seasons (2023 and 2024). Compared with the control treatment, the treatment with 10 mg L^− 1^ ZnO NPs + molasses consistently resulted in the highest TSS (16.42 and 16.26 brix), TSS/acid ratio (25.57 and 25.10), and berry firmness (229.74 and 238.59 g/mm), accompanied by a reduction in acidity (0.648 and 0.648%) in 2023 and 2024, respectively. Compared with ZnO NPs alone, ZnO NPs plus molasses had a greater effect, likely due to increased nutrient uptake, improved metabolic activity, and stimulation of biochemical pathways associated with fruit ripening and firmness.

**Table 3:** Effect of ZnO nanoparticles and molasses foliar application on the TSS, acidity, TSS/acid ratio and firmness of Crimson seedless grapes during two seasons (2023 and 2024)

Parameter	TSS (Brix)	Acidity %	TSS/Acid ratio	Firmness (g/mm)	TSS (Brix)	Acidity %	TSS/Acid ratio	Firmness (g/mm)
Treatments	First season (2023)				Second season (2024)			
Control (tap water)	11.06 ^e^ ± 0.36	0.754 ^a^ ± 0.007	14.75 ^e^ ± 0.62	212.55 ^c^ ± 0.05	11.30 ^e^ ± 0.22	0.748 ^a^ ± 0.004	15.12 ^d^ ± 0.40	213.00 ^c^ ± 0.15
1.5 cm/L Molasses (M)	12.44 ^cd^ ± 0.44	0.726 ^bc^ ± 0.008	17.19 ^cd^ ± 0.81	216.45 ^bc^ ± 0.12	12.54 ^cd^ ± 0.32	0.726 ^b^ ± 0.006	17.36 ^bcd^ ± 0.59	214.32 ^c^ ± 0.33
10 mg L^− 1^ ZnO NPs	15.77 ^a^ ± 0.08	0.660 ^f^ ± 0.001	24.01 ^a^ ± 0.16	222.98 ^abc^ ± 0.13	16.12 ^a^ ± 0.42	0.650 ^e^ ± 0.008	24.87 ^a^ ± 0.99	224.25 ^b^ ± 0.18
50 mg L^− 1^ ZnO NPs	13.51 ^bc^ ± 0.12	0.702 ^de^ ± 0.002	19.20 ^bc^ ± 0.24	221.94 ^abc^ ± 0.09	13.78 ^b^ ± 0.11	0.700 ^d^ ± 0.003	19.73 ^b^ ± 0.22	218.04 ^bc^ ± 0.10
100 mg L^− 1^ ZnO NPs	12.70 ^cd^ ± 0.73	0.718 ^cd^ ± 0.014	17.97 ^cd^ ± 1.56	218.99 ^abc^ ± 0.18	13.32 ^bcd^ ± 0.77	0.708 ^bcd^ ± 0.015	18.97 ^bc^ ± 1.58	219.44 ^bc^ ± 0.07
10 mg L^− 1^ ZnO NPs + M	16.42 ^a^ ± 0.17	0.648 ^f^ ± 0.003	25.57 ^a^ ± 0.40	229.74 ^a^ ± 0.31	16.26 ^a^ ± 0.16	0.648 ^e^ ± 0.002	25.10 ^a^ ± 0.38	238.59 ^a^ ± 0.21
50 mg L^− 1^ ZnO NPs + M	14.38 ^b^ ± 0.24	0.686 ^e^ ± 0.005	20.99 ^b^ ± 0.48	225.98 ^ab^ ± 0.24	13.66 ^bc^ ± 0.32	0.702 ^cd^ ± 0.006	19.53 ^bc^ ± 0.66	226.20 ^b^ ± 0.19
100 mg L^− 1^ ZnO NPs + M	11.69 ^de^ ± 0.39	0.740 ^ab^ ± 0.007	15.85 ^de^ ± 0.70	217.22 ^bc^ ± 0.31	12.84 ^d^ ± 0.17	0.724 ^bc^ ± 0.002	17.24 ^cd^ ± 0.32	224.16 ^b^ ± 0.43

Means in each column with the same letter(s) are not significantly different at the 5% level

### Grape biochemical components and PAL enzyme activity

#### Total anthocyanin content

**Fig. 2:**
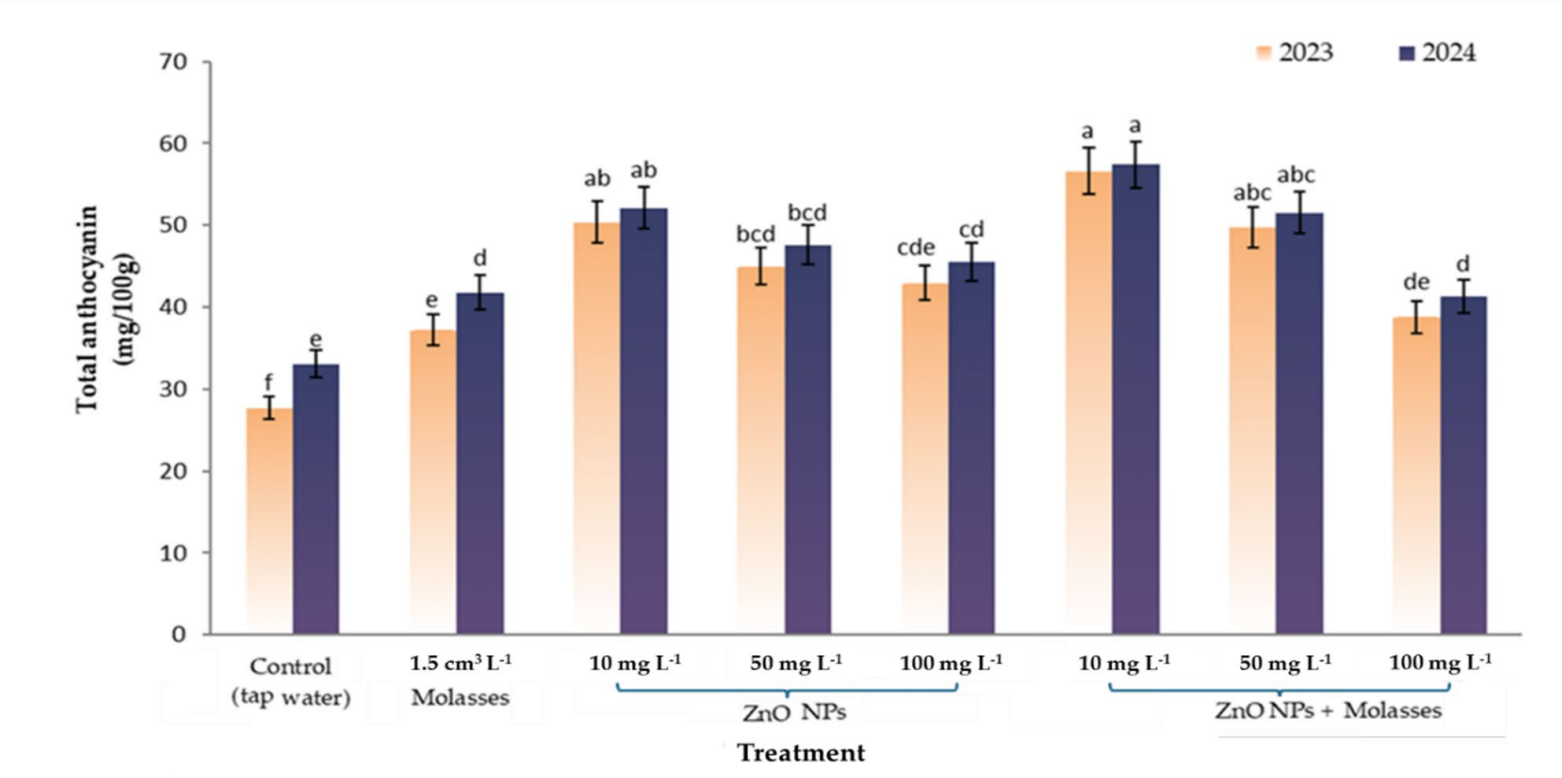
Effects of foliar application of ZnO nanoparticles and molasses on the total anthocyanin content in the berry skin of the Crimson seedless grape cultivar during two seasons: 2023 and 2024

The total anthocyanin content in the control berries reached 27.7 and 33.1 mg 100 g⁻¹ FW in both seasons, respectively, as shown in Fig. 2. The highest level of anthocyanin accumulation was observed with the 10 mg L-1 ZnO NPs + molasses treatment, with values of 56.6 and 57.4 mg/100 g in both seasons. Notably, lower concentrations of ZnO NPs were more effective in promoting anthocyanin accumulation in the berry skin. Additionally, the combined application of ZnO NPs and molasses resulted in the greatest anthocyanin accumulation, exceeding that in both the control and individual ZnO NP treatments.

#### PAL enzyme activity

**Fig. 3:**
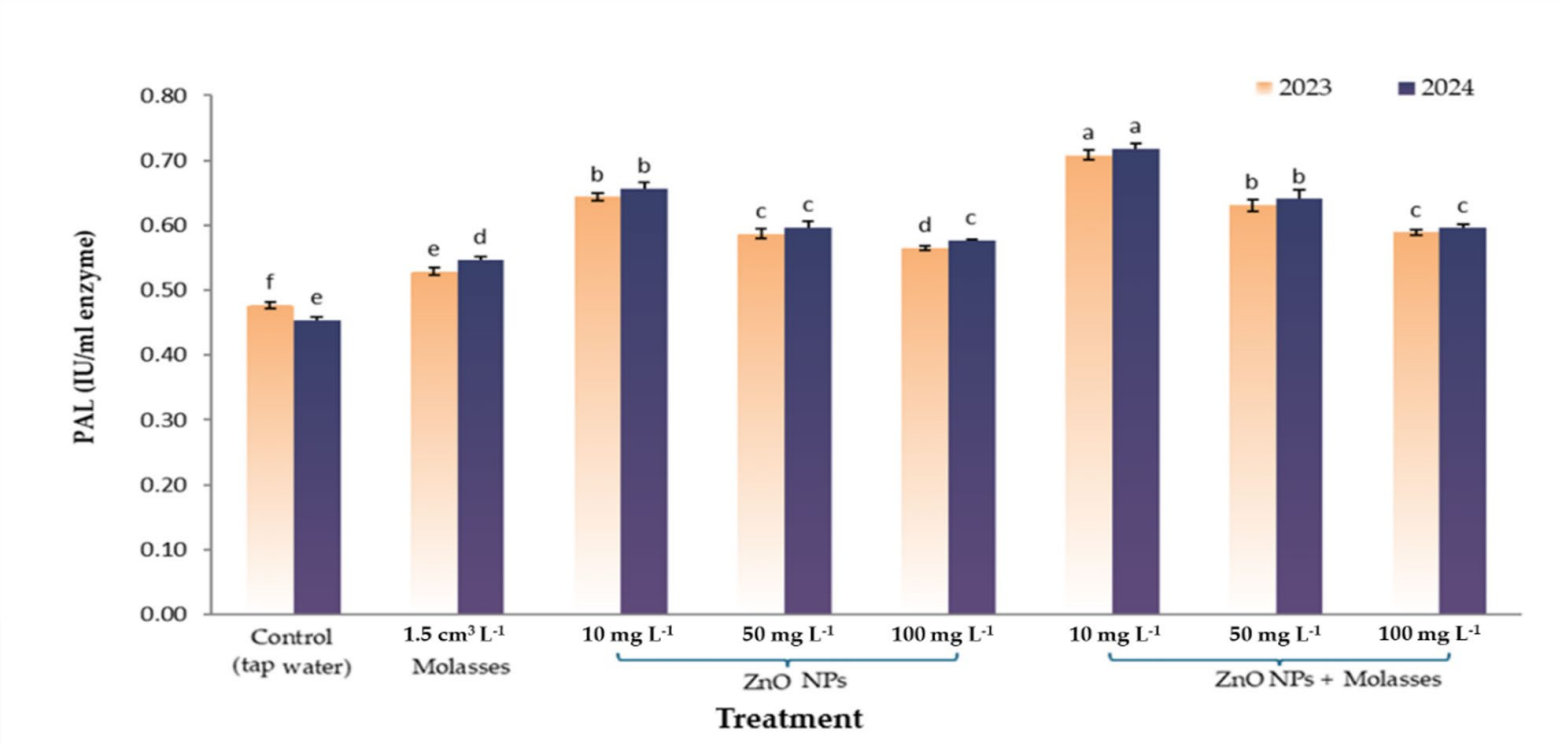
Effects of foliar application of ZnO nanoparticles and molasses on PAL enzyme activity in the berries of the Crimson seedless grape cultivar during two seasons: 2023 and 2024

The data shown in Fig. 3 revealed a significant increase in phenylalanine ammonia-lyase (PAL) activity expressed per unit volume of crude enzyme extract in grape clusters treated with ZnO nanoparticles, particularly when combined with molasses, compared with the control. In both seasons, the 10 mg L^− 1^ ZnO NPs + molasses treatment resulted in the highest PAL activity (0.708–0.718 IU/ml enzyme) in both seasons, respectively, followed closely by the 10 mg L^− 1^ ZnO NPs alone. Pearson correlation analysis between PAL activity and anthocyanin content. The correlation coefficient was computed via Pearson’s method in Excel. The results revealed a strong positive correlation (*r* = 0.834), indicating a likely direct and significant relationship between increased activity of the PAL enzyme and increased accumulation of anthocyanin pigment under the tested treatments.

### Colorimetric evaluation

**Fig. 4:**
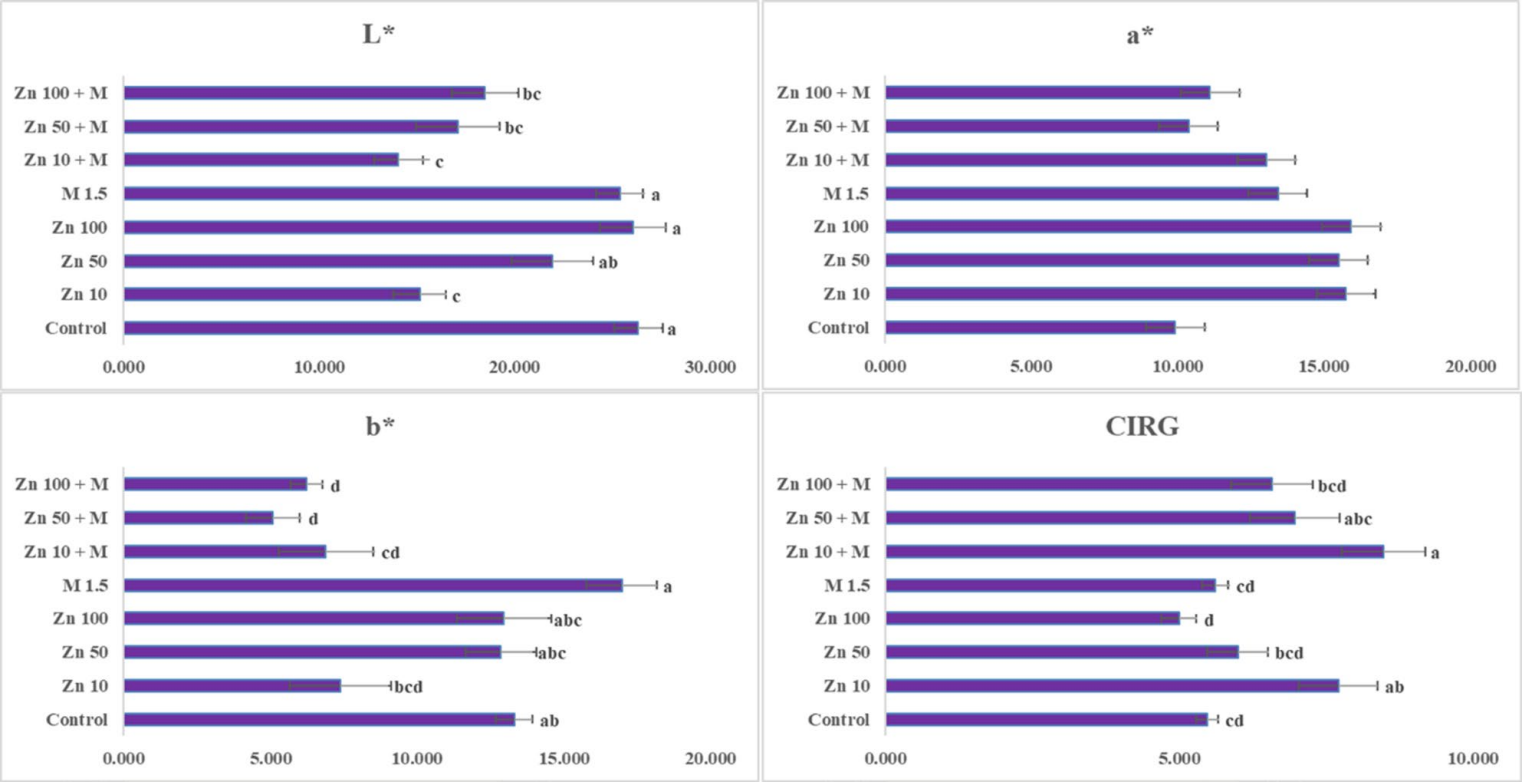
Effect of ZnO nanoparticles and molasses foliar application on berry color parameters (L*, a*, b*, and CIRG) in berries of the Crimson seedless grape cultivar. L*: lightness; a*: red–green axis; b*: yellow–blue axis; CIRG: color index of red grapes

**Fig. 5:**
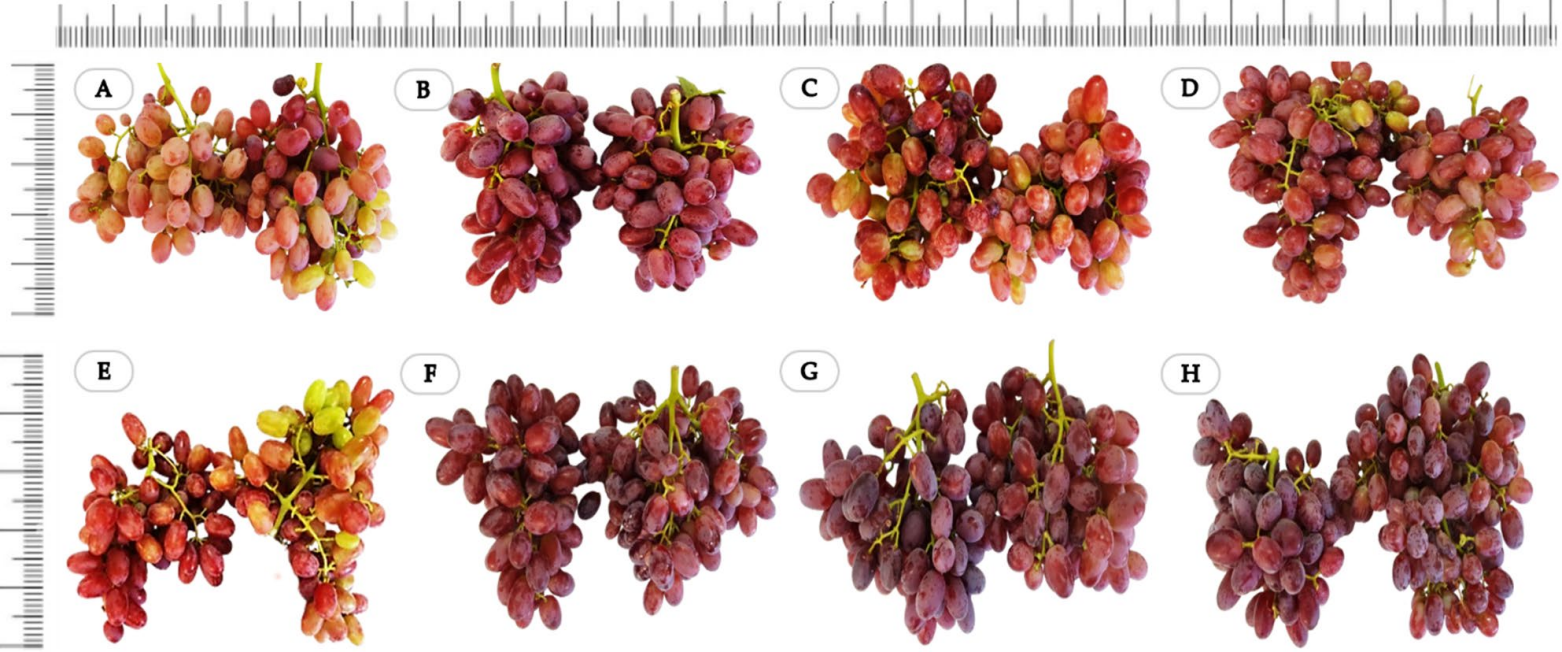
Appearance of ‘Crimson Seedless’ grape clusters treated with **(A)** the control (tap water), **(B)** 10 mg L^-1^ ZnO NPs, **(C**) 50 mg L^-1^ ZnO NPs, **(D)** 100 mg L^-1^ ZnO NPs, **(E)** 1.5 cm^3^ L^-1^ molasses, **(F)** 10 mg L^-1^ ZnO NPs + molasses, **(G)** 50 mg L^-1^ ZnO NPs + molasses, and **(H)** 100 mg L^-1^ ZnO NPs + molasses

A colorimetric evaluation of the grape berries revealed clear differences among the treatments (Fig. 4). The lightness (L*) ranged from 14.08 to 26.33, with the lowest values observed under 10 mg L^− 1^ ZnO NPs + molasses (14.08) and 10 mg L^− 1^ ZnO NPs (15.15), indicating markedly darker berries than those in the control (26.33). The redness index (a*) varied between 9.92 and 15.92, where all the nano-zinc treatments produced higher a* values than did the control (9.92), with the highest a* recorded in the 100 mg L^− 1^ ZnO NPs (15.92). The yellowness parameter (b*) clearly decreased under the combined treatments, decreasing from 16.97 under the molasses treatment and 13.31 under the control to 5.09 and 6.24 under the 50 mg L^− 1^ ZnO NPs + molasses and 100 mg L^− 1^ ZnO NPs + molasses treatments, respectively. This decline reflects a substantial reduction in yellow tones and a shift toward deeper red pigmentation. The color index for red grapes (CIRG) demonstrated the strongest treatment effect, ranging from 4.99 to 8.47. The highest CIRG value was recorded for 10 mg L^− 1^ ZnO NPs + molasses (8.47), followed by 10 mg L^− 1^ ZnO NPs alone (7.71), indicating an increased red color intensity. In contrast, the control (5.47) and molasses alone (5.61) had the lowest CIRG values. Overall, low-dose nano-zinc, particularly when combined with molasses, produced the most desirable increase in berry color by reducing L*, lowering b*, and markedly increasing CIRG, suggesting that the use of molasses with ZnO NPs could enhance anthocyanin synthesis and accumulation. These findings are illustrated by photos of the clusters at harvest (Fig. 5).

### Principal component analysis (PCA)

**Fig. 6:**
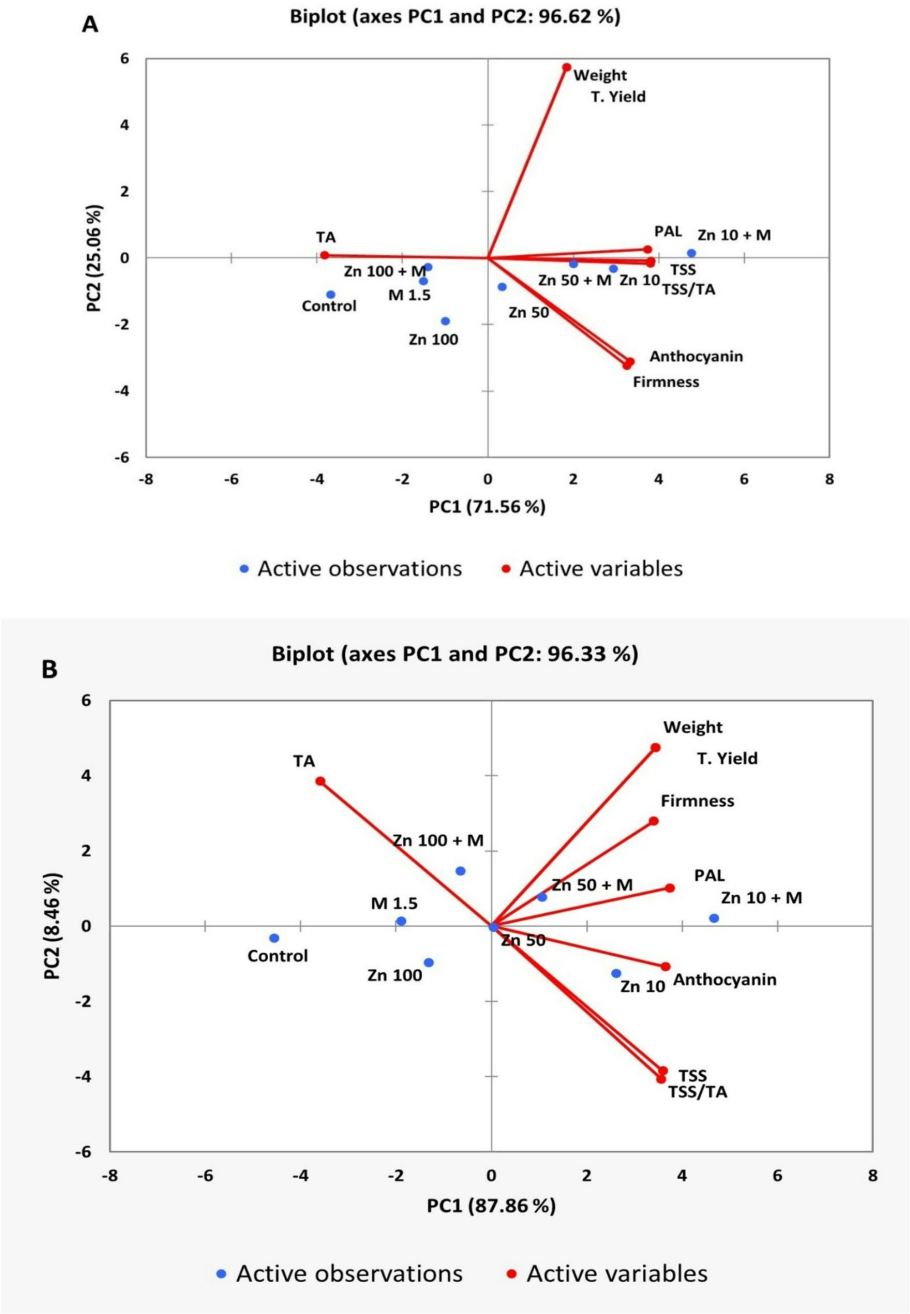
Principal component analysis (PCA) of ZnO NPs and molasses foliar sprays and variable trait relationships in the Crimson seedless grape cultivar via data obtained in 2023 **(A)** and 2024 **(B)**

A PCA biplot was used to summaries the relationships among the evaluated treatments of ZnO nanoparticle and molasses foliar application and the physical and biochemical fruit quality attributes of the grape cv. Crimson Seedless in both seasons, as shown in Fig. 6. PC1 explained most of the variation, with values of 71.56% and 87.86% in the two seasons, respectively. PC1 was positively correlated with most variables, whereas TA showed a negative correlation. PC2 represented 25.06% and 8.46% of the variance in the two seasons, respectively. ZnO NPs at 10 mg L^− 1^ + molasses and ZnO NPs at 50 mg L^− 1^ + molasses were positively associated with yield, weight, and PAL activity-related parameters, whereas ZnO NPs at 50 mg L^− 1^ and ZnO NPs at 10 mg L^− 1^ were associated with fruit quality traits such as the TSS, firmness, and anthocyanin content. In contrast, ZnO NPs at 100 mg L^− 1^ and the control group were located on the negative side of PC1, showing a weak association with most quality and yield traits. These summaries show that the combined application of ZnO NPs and molasses tends to enhance yield attributes, whereas moderate ZnO NPs doses alone favor quality-related parameters.

### Heatmap analysis

**Fig. 7:**
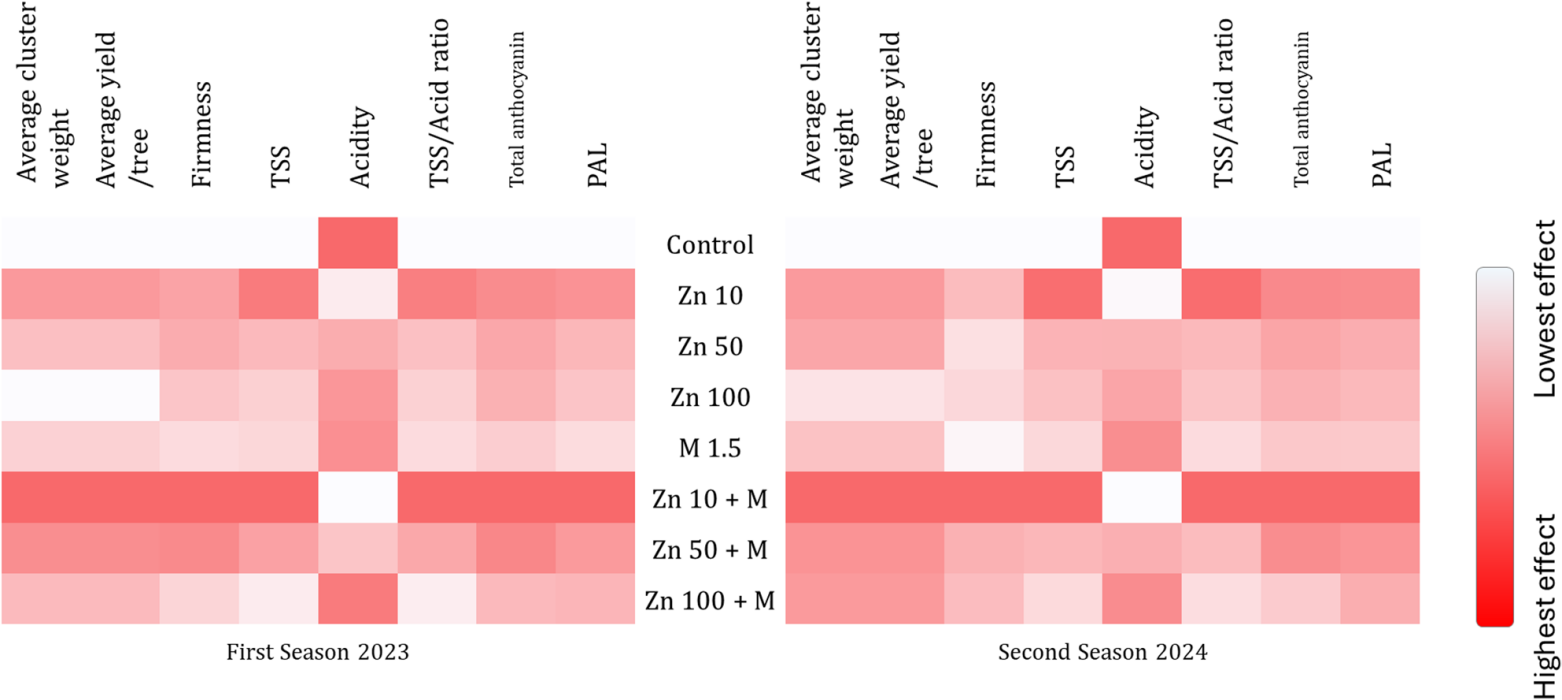
Heatmap of the effects of foliar spraying with ZnO NPs and molasses on various studied traits in the Crimson seedless grape cultivar during the 2023 and 2024 seasons

The heatmaps present a comparison of treatment-induced variations in grape quality and yield characteristics over two consecutive seasons, as shown in Fig. 7. Darker hues on a red gradient scale indicate higher values for the associated trait. The heatmap clearly shows differences between the treatments, with several ZnO NPs and ZnO NPs + molasses treatments resulting in higher intensities for fruit-quality characteristics such as TSS, anthocyanins, and PAL in both seasons. For most traits, however, the control treatment had relatively low intensities. Overall, these patterns suggest that lower ZnO NP concentrations, particularly when combined with molasses, are associated with improvements in multiple yield and quality traits, with minor variations between seasons.

## Discussion

The external appearance of grape clusters, particularly berry coloration, plays a fundamental role in determining the marketability and consumer preference for table grapes [73], as fruit color is a key indicator of maturity, freshness, and quality [74]. Although the present study did not directly quantify molecular regulators, the coordinated increase in PAL activity, anthocyanin accumulation, CIRG values, and sugar–acid balance provides supportive physiological indications that may be consistent with the activation of the phenylpropanoid and flavonoid biosynthetic pathways [75]. PAL is a rate-limiting enzyme in anthocyanin synthesis, and its significant stimulation under low-dose ZnO NPs treatments suggests a possible link between the observed improvements in berry coloration and the underlying biochemical regulation of secondary metabolism; however, this interpretation remains tentative and requires further validation through targeted molecular or biochemical analyses. The development of grape berries involves a complex series of physical and biochemical changes, including adjustments to size, chemical composition, color, texture, and flavor [76]. Various horticultural practices, such as leaf removal, topping, and bunch thinning, decrease mutual shade. However, these viticultural practices are often insufficient to address the poor color of ‘Crimson Seedless’ grape cultivars. Exogenous applications of ZnO NPs have been shown to increase grape cluster quality [14, 77, 78]. Additionally, the addition of bioproducts such as molasses could increase the physiological response of plants [79]. The integration of ZnO nanoparticles with molasses could play a critical role in mitigating the negative effects of climatic fluctuations on berry coloration and grape quality. Nanoparticles provide essential zinc in a highly available form that supports enzymatic activity, anthocyanin biosynthesis, and antioxidant defense systems [80], whereas molasses supplies organic carbon and essential nutrients [81, 82], thereby potentially increasing leaf metabolic activity. This dual action can improve the physiological performance of grapevines, leading to more uniform skin coloration, higher cluster weight, and improved marketability of the Crimson Seedless grape cultivar.

Zinc plays both direct and indirect roles in the synthesis of tryptophan and auxins [83–85]. Although ZnO nanoparticles exhibit low bulk water solubility, their biological effectiveness does not depend solely on complete dissolution. Nano-sized ZnO particles have been reported to interact with leaf surfaces and potentially penetrate through stomatal openings or nano-scale pores [86, 87]. In addition, nanoparticles may function as metabolic or signaling modulators, eliciting physiological responses disproportionate to their applied mass. The intercellular transport of auxin influences a variety of growth and developmental processes, such as cell division and elongation [88]. Zn can improve berry quality, which in turn affects the cluster quality and overall yield of Crimson Seedless grapevines, as it acts as a co-factor in the synthesis of amino acids and proteins needed for cell division, cell enlargement, and cell differentiation [89]. The relative improvement in the weight of clusters sprayed with ZnO NPs is in accordance with the findings of [90], who clearly showed that foliar spraying with Zn NPs at 120 g L^-1^ plus B NPs at 6.5 g L^-1^ resulted in the highest fruit weight of pomegranate trees. Additionally [77, 78], reported that foliar spraying with nano-Zn resulted in the highest cluster weight and dimensions and led to significant increases in the cluster physical and berry parameters of the Flame Seedless grape cultivar.

Furthermore, the combination of nanoparticles with organic bioproducts may contribute to improving yield stability and enhancing fruit nutritional quality, although their effects on residue accumulation and environmental safety remain to be fully elucidated. ZnO nanoparticles are generally regarded as relatively safe materials and are widely used in various consumer products; however, their safety in agricultural applications depends on concentration, exposure level, and environmental conditions. According to the U.S. Food and Drug Administration (FDA), ZnO is classified as a substance generally recognized as safe for certain uses [40, 41] however, this classification does not necessarily extend to all agricultural applications or guarantee the absence of potential risks under field conditions. Previous studies have reported that nanomaterials, when applied at low concentrations, act as efficient biostimulants that increase pigment formation on fruit skin, sugar accumulation, and antioxidant activity in fruits [14, 45]. Similarly, molasses supplementation has been shown to improve the chlorophyll content, photosynthetic activity, and crop yield of a variety of plants, which suggests its potential as a complementary input in viticulture [58, 79]. Molasses acts as a bio-stimulant and energy source and supports nutrient assimilation [91]. Molasses-treated plants, such as sugar beet [62] and sweet pepper [92], presented significant increases in growth and productivity. Compared with molasses alone, molasses combined with ZnO nanoparticles had a greater effect, leading to greater improvements in grape cluster weight and total yield.

The application of ZnO NPs at 10 mg L^-1^ supplemented with molasses as a foliar spray improved the TSS of Crimson Seedless grape berries, reduced their acidity, and increased their berry firmness. In addition to preserving the structural stability of cell membranes, zinc may play a role in the synthesis and translocation of proteins and carbohydrates, which could be the reason for these effects [46, 47]. Additionally, zinc is essential for several metabolic processes [93], and once fruits mature, their organic acid content decreases by dissimilating or metabolizing stored organic acids [94, 95]. The results of [96] were in line with the results of the present study, as they reported that Zn improved the total soluble solids and reduced the total acidity of grape cultivars. The added effect of molasses may be attributed to its richness in readily available sugars and organic compounds, which act as a carbon source [97] that improves nutrient absorption and provides additional energy for metabolic processes. These mechanisms likely increase the efficiency of zinc uptake and utilization, thereby amplifying its role in protein and carbohydrate metabolism, membrane stability, and enzymatic regulation. Consequently, the combined treatment with ZnO NPs and molasses resulted in greater improvements than either treatment alone.

Interestingly, lower concentrations of ZnO NPs were more effective than higher concentrations at increasing anthocyanin accumulation in berry skins. The greater effectiveness of low ZnO NPs concentrations than higher doses may be interpreted within the framework of hormesis theory, which describes a biphasic dose–response relationship in which low levels of a stimulus enhance biological performance, whereas excessive concentrations may induce metabolic stress [98, 99]. This may explain the optimal performance observed at 10 mg L^-1^ in the present study. Compared with the control, molasses treatment alone also increased the anthocyanin content, although its effect was lower than that of ZnO NPs treatments. A clear effect was observed when nano-zinc was combined with molasses, which resulted in greater improvements than either treatment alone. These differences were visually confirmed by the appearance of clusters at harvest. The intensity and uniformity of coloration are primarily determined by anthocyanin accumulation. This process is regulated by multiple factors, including genetic background, environmental conditions, cultural practices, and plant nutritional status [100]. Anthocyanins accumulate predominantly in the skin of most pigmented grape varieties during berry ripening [101], and biosynthesis occurs in red berry skins through the flavonoid pathway, starting with the precursor phenylalanine [102]. Anthocyanin accumulation is affected by various factors, including plant hormones and environmental conditions, both of which regulate anthocyanin biosynthesis through complex signal transduction and transcriptional regulatory systems [103–105]. Several key enzymes are involved in the synthesis of anthocyanins, including phenylalanine ammonia lyase (PAL), cinnamate 4-hydroxylase (CH), 4-coumaroyl-CoA ligase (4CL), chalcone synthase (CHS), chalcone isomerase (CHI), flavanone 3-hydroxylase (F3H), flavonoid 3’-monooxygenase (F3’H), flavonoid 3’,5’-hydroxylase (F3’,5’H), dihydroflavonol 4-reductase (DFR), anthocyanidin synthase (ANS), and UDP glucose-flavonoid glucosyltransferase (UFGT) [106]. The observed changes in PAL activity align with earlier reports demonstrating that enzyme pathways in plants are highly responsive to external nutritional or environmental stimuli [30, 37, 107]. More detailed mechanistic validation, including protein-normalized enzyme assays, gene expression analysis (e.g., PAL, CHS, UFGT), and replicate-level correlations, would be required to confirm a direct regulatory role.

## Conclusion

Under the specific conditions of this experiment, the combined foliar application of low‑dose ZnO nanoparticles (10 mg L^− 1^) with 1.5 cm³ L⁻¹ molasses has practical potential for improving berry color, firmness, and soluble solids in Crimson Seedless grape cultivar, which were grafted on Richter 110 rootstock. Biochemically, dual application stimulated phenylalanine ammonia-lyase (PAL) activity and anthocyanin accumulation, resulting in darker, more intensely red berries with higher color index values (CIRG). Low concentrations of ZnO NPs were more effective than higher doses under the tested conditions. For growers facing uneven coloration or suboptimal fruit quality under the same conditions of this experiment, a single application at veraison may provide a simple and simple, cost-effective approach to enhance marketability. These findings highlight the potential of combining nanotechnology with organic stimulants as a sustainable approach for improving grapevine performance while reducing reliance on conventional chemical inputs. Further multi‑site, multi‑season studies involving additional cultivars, rootstocks, and application timings are necessary to confirm consistency of response. In addition, long‑term assessments of ZnO nanoparticle behavior, environmental safety, and residue dynamics in vineyard systems are required before broader agronomic recommendations can be made.

## Data Availability

The datasets generated during the current study are available from the corresponding author on reasonable request.
